# Analysis of Fundus Shape in Highly Myopic Eyes by Using Curvature Maps Constructed from Optical Coherence Tomography

**DOI:** 10.1371/journal.pone.0107923

**Published:** 2014-09-26

**Authors:** Masahiro Miyake, Kenji Yamashiro, Yumiko Akagi-Kurashige, Akio Oishi, Akitaka Tsujikawa, Masanori Hangai, Nagahisa Yoshimura

**Affiliations:** Department of Ophthalmology and Visual Sciences, Kyoto University Graduate School of Medicine, Kyoto, Japan; University of Melbourne, Australia

## Abstract

**Purpose:**

To evaluate fundus shape in highly myopic eyes using color maps created through optical coherence tomography (OCT) image analysis.

**Methods:**

We retrospectively evaluated 182 highly myopic eyes from 113 patients. After obtaining 12 lines of 9-mm radial OCT scans with the fovea at the center, the Bruch’s membrane line was plotted and its curvature was measured at 1-µm intervals in each image, which was reflected as a color topography map. For the quantitative analysis of the eye shape, mean absolute curvature and variance of curvature were calculated.

**Results:**

The color maps allowed staphyloma visualization as a ring of green color at the edge and as that of orange-red color at the bottom. Analyses of mean and variance of curvature revealed that eyes with myopic choroidal neovascularization tended to have relatively flat posterior poles with smooth surfaces, while eyes with chorioretinal atrophy exhibited a steep, curved shape with an undulated surface (P<0.001). Furthermore, eyes with staphylomas and those without clearly differed in terms of mean curvature and the variance of curvature: 98.4% of eyes with staphylomas had mean curvature ≥7.8×10^−5^ [1/µm] and variance of curvature ≥0.26×10^−8^ [1/µm].

**Conclusions:**

We established a novel method to analyze posterior pole shape by using OCT images to construct curvature maps. Our quantitative analysis revealed that fundus shape is associated with myopic complications. These values were also effective in distinguishing eyes with staphylomas from those without. This tool for the quantitative evaluation of eye shape should facilitate future research of myopic complications.

## Introduction

Myopia is one of the most common visual disorders worldwide, representing a major public health concern among East Asian populations. [1]–[4] Myopic eyes with very long axial lengths (≥26.0 mm, ≥26.5 mm, or ≥28.0 mm) or a high degree of myopic refractive error (≤−5D, ≤−6D, or ≤−8D) are classified as highly myopic. [5]–[11] High myopia is typically associated with excessive elongation of the globe and, in middle-aged individuals, often leads to pathological changes such as posterior staphyloma. [12], [13] Moriyama et al. analyzed the shapes of eyes with pathologic myopia using three-dimensional magnetic resonance imaging (MRI). The results demonstrated a clear association between eye shape and the likelihood of optic neuropathy. [14] However, eyeball shape as classified by MRI was not associated with myopic complications at the posterior pole such as myopic choroidal neovascularization (mCNV), chorioretinal atrophy (CRA), or myopic tractional maculopathy. Owing to the resolutional limitations of MRI, this technique cannot be used for detailed morphological investigations of the posterior pole, where most major myopic complications develop.

Today, optical coherence tomography (OCT) is emerging as a popular modality by which we can obtain precise cross-sectional images of retina and fundus, though its scan width is limited. While MRI is a very useful device with which to macroscopically evaluate eyeball shape, [14]–[16] OCT is more suitable for microscopic evaluation owing to its high resolution. The rapid scanning speed utilized for spectral-domain OCT can minimize the effects of ocular movement, allowing for a more precise analysis of retinal structure. [17] To date, several studies have examined staphylomas using OCT. [18], [19] Notably, the height of a posterior staphyloma affects the development of mCNV. Previous reports investigated this phenomenon using several narrow 6-mm scans, which collectively captured only a small portion of the entire staphyloma. [18] In the current study, we evaluated twelve 9-mm radial OCT scans in each highly myopic eye studied. This information was used to create a curvature map for the nearly entirety of Bruch’s membrane; the staphyloma edge and degree of staphyloma were clearly visualized with the aid of this map. Subsequent analyses revealed associations between posterior pole shape and myopic complications.

## Materials and Methods

We examined 192 eyes respectively, having axial length ≥26 mm from 120 consecutive patients who underwent the analysis below during the period from April 2010 to March 2012. All procedures in this study adhered to the tenets of the Declaration of Helsinki. The ethics committee (Ethics Committee of Kyoto University Graduate School and Faculty of Medicine, Japan) approved the study protocol. All of the patients were fully informed about the purpose and procedures of this study, and written consent was obtained from each. Patient records/information was anonymized and de-identified prior to analysis.

### Data collection

Of the 192 eyes, 10 eyes from 7 patients were excluded either because the OCT images and/or color fundus photographs were of poor quality or because they had a history of ocular surgery other than cataract surgery. A total of 182 highly myopic eyes from 113 patients were analyzed. Each subject underwent a complete ophthalmic examination, including axial length measurement using an IOL master (Carl Zeiss Meditec, Dublin, CA), indirect ophthalmoscopy and slit-lamp biomicroscopy, and optical coherence tomography (RS-3000, Nidek, Tokyo, Japan). Fluorescein angiography was performed if mCNV was suspected.

### Mapping fundus curvature

In this analysis, we used Burch’s membrane lines as representative lines of the fundus shape instead of the retina or RPE lines, which allowed us to overcome the drawbacks of previous method using retinal topography [20], [21]; retinal or RPE topography can be easily biased by complications such as CNV, schisis, and MHRD.

**Figure 1:**
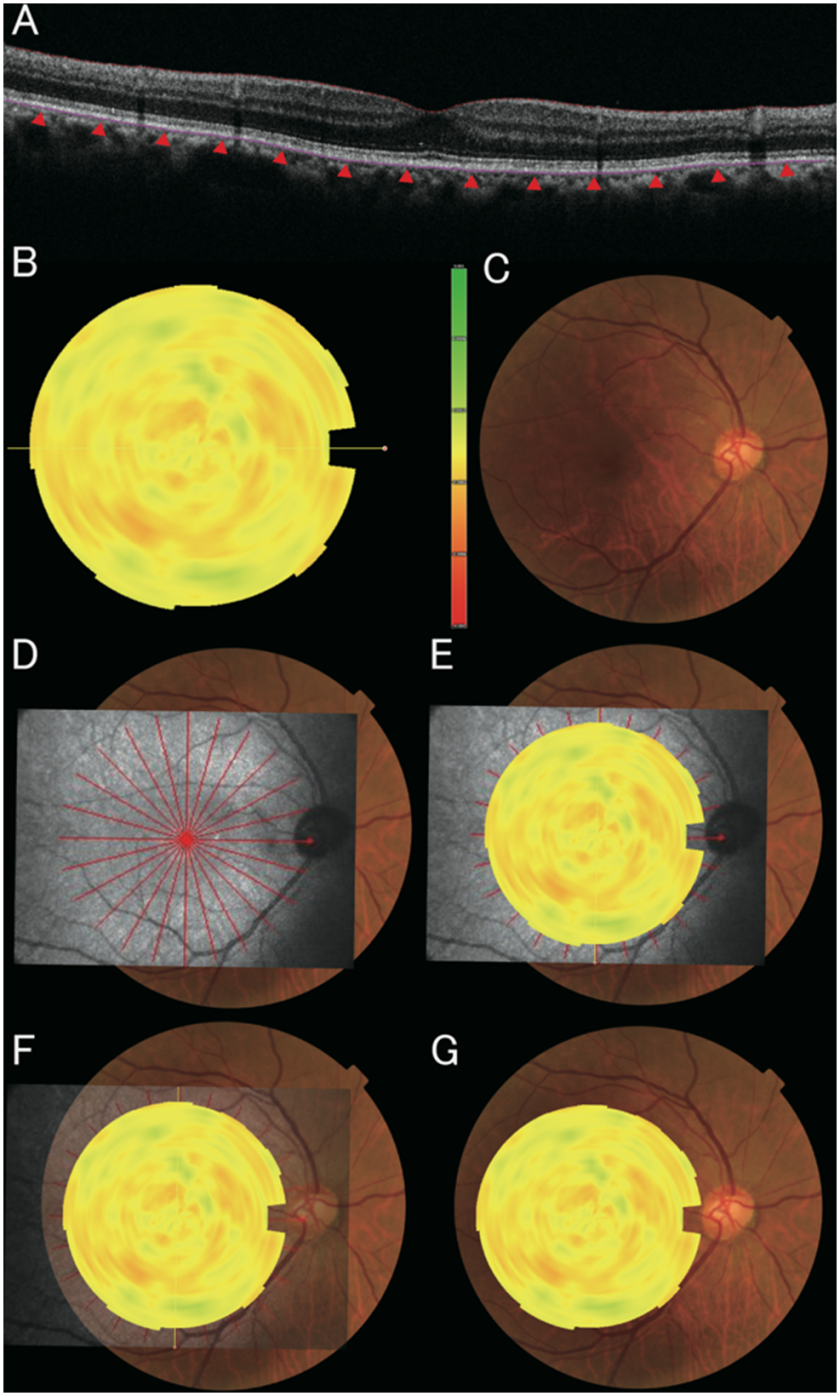
Construction of the curvature map for a normal eye. **A,** Bruch’s membrane was plotted in each of 12 radial OCT scans with the fovea at the center. Local curvature was measured from 3 sequential points (sampled at 500-µm intervals). The sine of curvature was defined as positive when the membrane was convex upward. **B,** The value of curvature was mapped using yellow (RGB(255, 255, 0)) to represent zero curvature (flat), green (gradient from RGB(255, 255, 0) to RGB(0, 192, 32) according to curvature [1/µm]) for positive curvature (convex-upward), and red (gradient from RGB(255, 255, 0) to RGB(255, 0, 0) according to curvature [1/µm]) for negative curvature (convex-downward). **C,** Color fundus photograph on which the topographic maps will be overlaid. **D,** Scanning Laser Ophthalmoscope (SLO) images are overlaid on the fundus photographs. **E,** Topographic maps are overlaid on the SLO images. **F and G,** SLO image transparency was increased for an accurate superimposition.



We built new software to calculate the eye curvatures from their OCT images and construct a color curvature map. This software presents a measure of retinal shape with distribution of local curvature. By calculating the local curvature, it was possible to detect small unevenness of the retina. After obtaining 12 lines of a 9-mm radial OCT scan at center of the fovea, Bruch’s membrane line was plotted (Figure 1A). The size of each OCT image was adjusted to correct for the difference in pixel resolution in the transverse and longitudinal directions. A case in which a curvature of the Bruch’s membrane was used as shape analysis is described below using the OCT image shown in Figure 1. The curvature of the Bruch’s membrane line as an analysis target was calculated. The curvature κ can be obtained by calculating, at respective points at the Bruch’s membrane line:

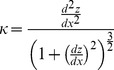
(1)The differentials in equation (1) were calculated by central difference. These equations are represented by:
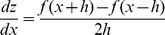
(2)

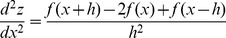
(3)where f(x) is the Bruch’s membrane line, and h is the spacing between two adjacent A-scans (500-µm apart). The curvature κ was measured using 3 sequential A-scans. Therefore, it represents local shape in the range of 1,000-µm (Local curvature). The values of the local curvature were calculated using all A-scans except in regions within 500-µm from right and left boundaries of an image. The unit of curvature κ was inverse micrometer. Positive, negative, or zero value of the curvature κ represents upward concave, downward convex, or flat respectively. In this study, the curvature was calculated with three points on the Bruch’s membrane line. Because distance from the first point to the third point was 1000-µm, this localized curvature was allowed to be zero.

**Figure 2:**
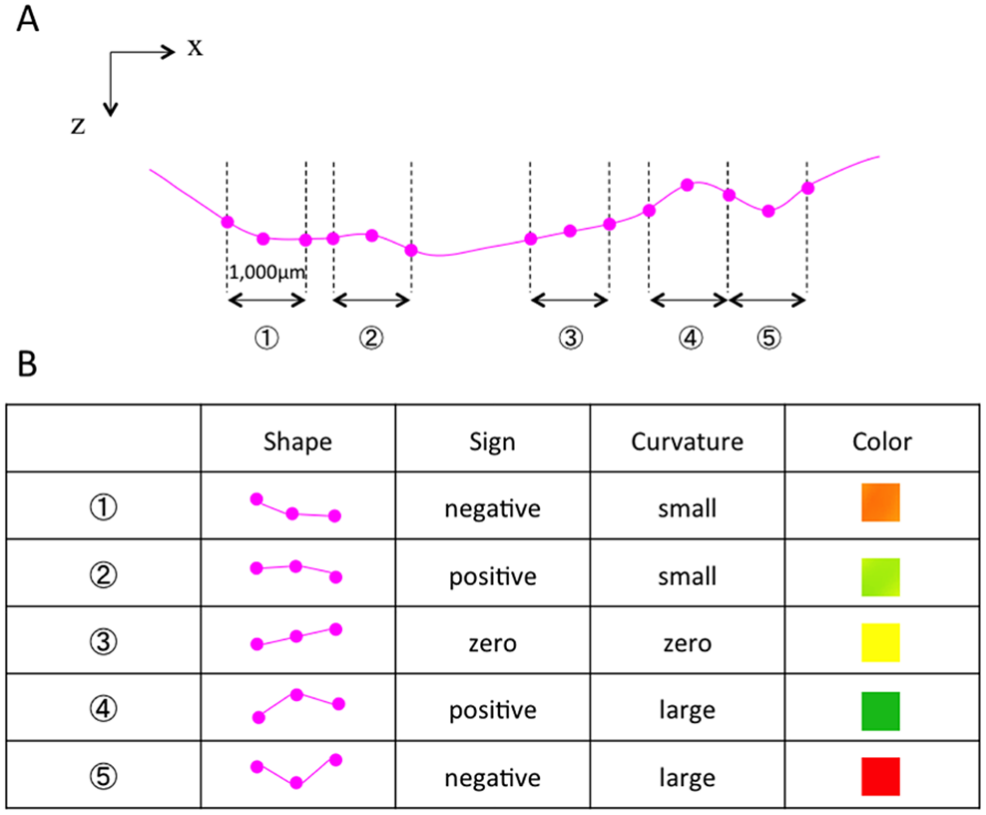
Image of correspondence between fundus shape, curvature, and color. **A,** An imaginary Bruch’s membrane line to exemplify the correspondence. The curvature of one point was measured using 3 sequential A-scans (500-µm apart) so that it represented local shape in the range of 1,000-µm (Local curvature). Five points are exemplified in this figure. **B,** Corresponding curvature [1/µm] and color image for each 5 point. Positive, negative, or zero value of the curvature κ represents upward concave, downward convex, or flat, respectively. The color of curvature map showed a gradient from green (RGB(0, 192, 32)) to yellow (RGB(255, 255, 0)) as a local curvature value changes from 0.0005 to 0.0, and a gradient from red (RGB(255, 0, 0)) to yellow (RGB(255, 255, 0)) as a local curvature value changes from −0.0005 to 0.0.

The degree of curvature was measured for the radial OCT scans with the fovea at the center. The distribution of the local curvature was presented as a color map overlaid on a c-scan retinal image: yellow (RGB(255, 255, 0)) for the local curvature value of 0.0 (1/infinity) [1/µm], green (RGB(0, 192, 32)) for the local curvature value of +0.0005 (1/2000) [1/µm], and red (RGB(255, 0, 0)) for the local curvature value of −0.0005 (1/2000) [1/µm]. The color of curvature map was limited to 1/2000 [1/µm]. The curvature map set a color to show a gradient from green to yellow as a local curvature value changes from 0.0005 to 0.0, and a gradient from red to yellow as a local curvature value changes from −0.0005 to 0.0. Examples of correspondence between fundus shape, curvature, and color were presented in Figure 2. Generated color map was then superimposed on a fundus photo, being intermediated by a Scanning Laser Ophthalmoscope (SLO) image (Figure 1B–1F). Figure 1G depicts a normal eye. Considering the spherical shape of a normal eye, the curvature of its posterior pole should be negative. However, its curvature is very low so that the map shows an overall yellow appearance.

### Segmentation Method

A smoothing filter (Median filter) was applied to OCT images to remove noise components. Layer boundary extraction could be implemented by edge detection filter (Sobel filter) and the layer structure-emphasized filter (Hessian filter).

As for the inner limiting membrane, a peak of the layer structure emphasizing filter was searched for in the depth direction of fundus from the vitreum side, and a first peak position equal to or larger than a threshold was detected as an initial value of the inner limiting membrane and nerve fiber layer boundary. From that initial value, a gradient feature was searched for toward the vitreum side, and a gradient peak position was detected as the inner limiting membrane.

Likewise, as for the nerve fiber layer boundary, a gradient feature was searched for from that initial value in the depth direction of the fundus, and a gradient peak position was detected as the nerve fiber layer boundary.

As for the Bruch’s membrane line, a peak of the layer structure emphasizing filter is searched for further in the depth direction of the fundus, and the last peak position equal to or larger than the threshold is detected as an initial value of the Bruch’s membrane line. A gradient feature was searched for from that initial value in the depth direction of the fundus, and a gradient peak position was detected as the Bruch’s membrane line.

By applying Snakes [22] using the detected boundary as the initial position, the boundary shape became smooth.

### Data analysis

We evaluated the curvature maps for eyes with foveal retinal detachments (RDs), retinoschisis, mCNV, and severe CRA. In addition, differences in the curvature maps were compared between eyes with staphyloma and eyes without staphyloma. The presence of retinoschisis, RD, CRA, and staphyloma was determined using color fundus photographs and OCT images. FA images were used to identify mCNV. The images of each eye were examined by 2 retina specialists (K.Y and A.T), and any discrepancies were settled by a third specialist (N.Y). Cases showing patchy CRA >3 disc diameters in area were diagnosed as having severe CRA. The eyes with both retinoschisis and RD were classified as RD. Multiple comparisons of numerical values such as patient age and axial length were performed with analysis of variance (ANOVA) and Tukey–Kramer’s tests. Single comparisons were performed using an unpaired *t*-test. The male:female ratio and other 2×2 or 2×3 tables were compared using Fisher’s exact test and Tukey’s test for post-hoc analysis. A p-value of ≤5% was considered statistically significant.

## Results

Among these 113 patients, 37 were men and 76 were women, with a mean age of 61.9±14.1 years. The mean axial length was 28.68±1.74 mm. RD with or without retinoschisis was seen in 13 eyes (7.1%), retinoschisis without RD in 34 eyes (18.7%), mCNV in 33 eyes (18.1%), and severe CRA in 20 eyes (11.0%). The highly myopic patients with retinoschisis, RD, and severe CRA were significantly older than the highly myopic patients without these 3 complications (P = 1.6×10^−4^, 0.0017, 0.0012, respectively). There were more women than men with mCNV, retinoschisis, and CRA (Table 1). Axial length was significantly longer in eyes with retinoschisis, RD, and CRA as compared to eyes without complications, while the axial length of eyes with mCNV was not significantly different from eyes without these 4 complications.

**Table 1:** Characteristics of the included eyes according to complications.

Variable		No complications	mCNV	Retinoschisis	RD	CRA	P-value†
n		88	33	34	13	20	
Age (years ± SD)		56.2±13.9	60.3±16.1	67.9±11.9	71.0±8.56	68.9±6.51	1.16×10^−6^
p-value*	No complications	-	0.54	<0.001	0.002	0.001	
	mCNV	-	-	0.13	0.10	0.14	
	Retinoschisis	-	-	-	0.95	1.00	
	RD	-	-	-	-	0.99	
Sex (male:female)		44∶44	5∶28	6∶28	4∶9	3∶17	2.03×10^−4^
p-value*	No complications	-	0.002	0.004	0.60	0.02	
	mCNV	-	-	1.00	0.82	1.00	
	Retinoschisis	-	-	-	0.90	1.00	
	RD	-	-	-	-	0.86	
Axial length (mm ± SD)		28.02±1.49	28.35±1.20	29.46±1.54	29.31±1.38	30.96±1.11	5.47×10^−11^
p-value*	No complications	-	0.82	<0.001	0.04	<0.001	
	mCNV	-	-	0.03	0.31	<0.001	
	Retinoschisis	-	-	-	1.00	0.05	
	RD	-	-	-	-	0.11	

*Tukey-Kramer's multiple comparison.

† Analysis of variance.

CNV: choroidal neovascularization, RD: retinal detachment, CRA: chorioretinal atrophy.

### Constructing fundus curvature maps

**Figure 3:**
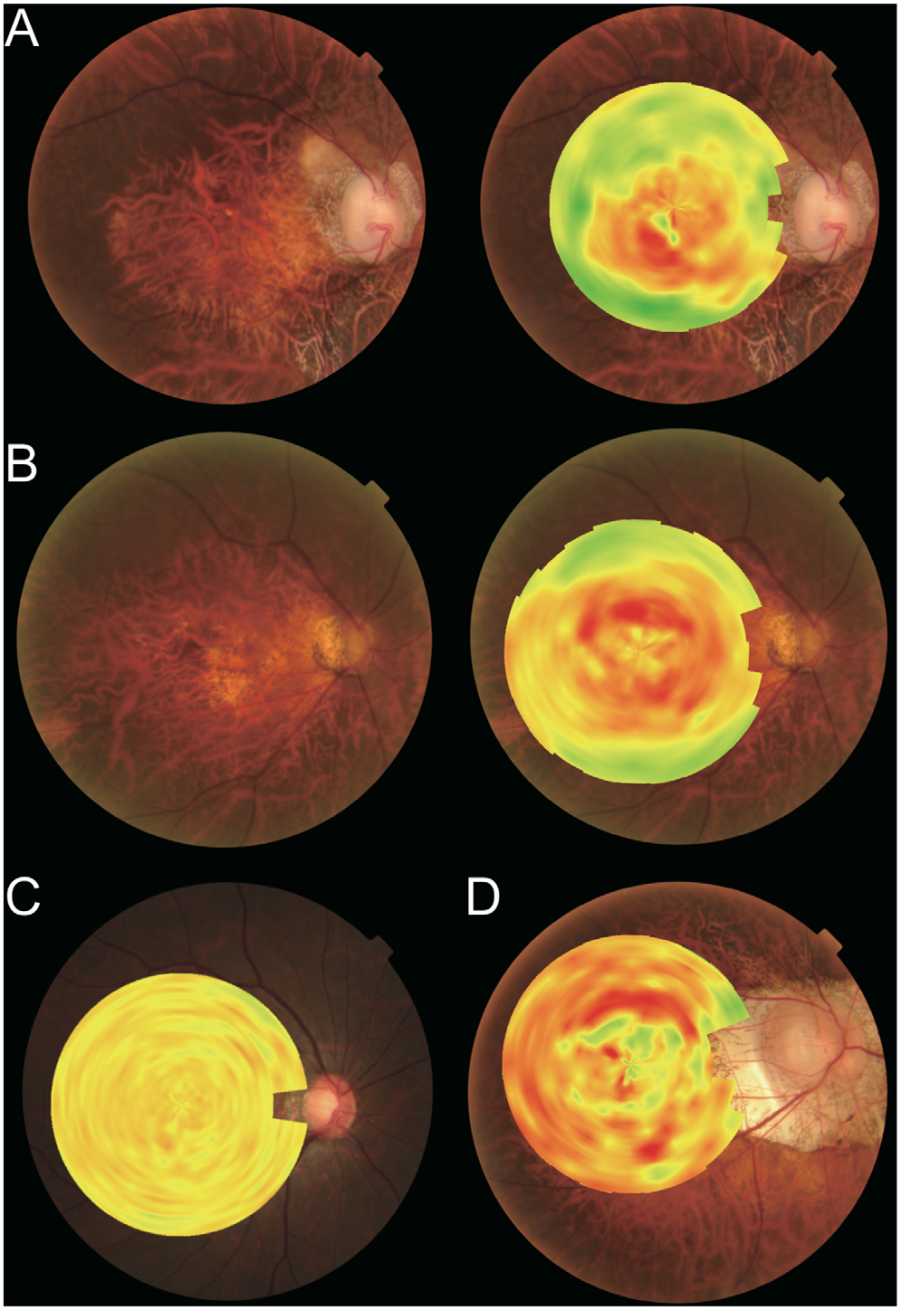
Representative color maps of highly myopic eyes. **A,** Right eye from a 58-year-old woman with axial length of 29.01 mm. The staphyloma edge is depicted in green-yellow. **B,** Right eye of a 72-year-old woman with axial length of 28.37 mm. Upper and lower edges of the staphyloma can be visualized clearly. **C,** Right eye of a 38-year-old woman, with axial length of 26.38 mm. The yellow-dominant color map represents a relatively flat fundus. **D,** Right eye from a 65-year-old woman with axial length of 34.64 mm. The mosaic color pattern indicates an undulated fundus.

Fundus curvature maps were successfully obtained and superimposed on color fundus photographs in all 182 cases. In 4 eyes with staphylomas, the entire staphyloma edge could be visualized clearly using this method (Figure 3A and 3B). Because the staphyloma edge must have an inflection point, the edge curvature theoretically ranges from zero to plus, which would be represented on the curvature map as yellow to green shading along the staphyloma edge. The area inside the staphyloma is depicted as red to orange, which represents a relatively high degree of concave curvature. This image can be contrasted with that of a representative normal eye, which yielded a topographical map that was yellow in overall appearance (Figure 1).

In this analysis, however, manual correction was needed in all cases more or less. While we required re-plot of less than 10 points per slice in eyes without staphyloma, re-plot of more than or equal to 10 points per slice was needed in most eyes with staphyloma to obtain correct curvature of Bruch’s membrane. When image inversion towards the edges of the OCT scan existed, we could not construct curvature map at the corresponding area. In the studied eyes, however, mean coverage area was 86.4±10.2%, which corresponds to 87.3%–98.3% coverage of diameter.

Some of the participants underwent several OCT examinations within 3 months. In these eyes, the color map did not show notable change within 3 months, suggesting our method has acceptable repeatability.

### Quantitative evaluation of the curvature maps

**Figure 4:**
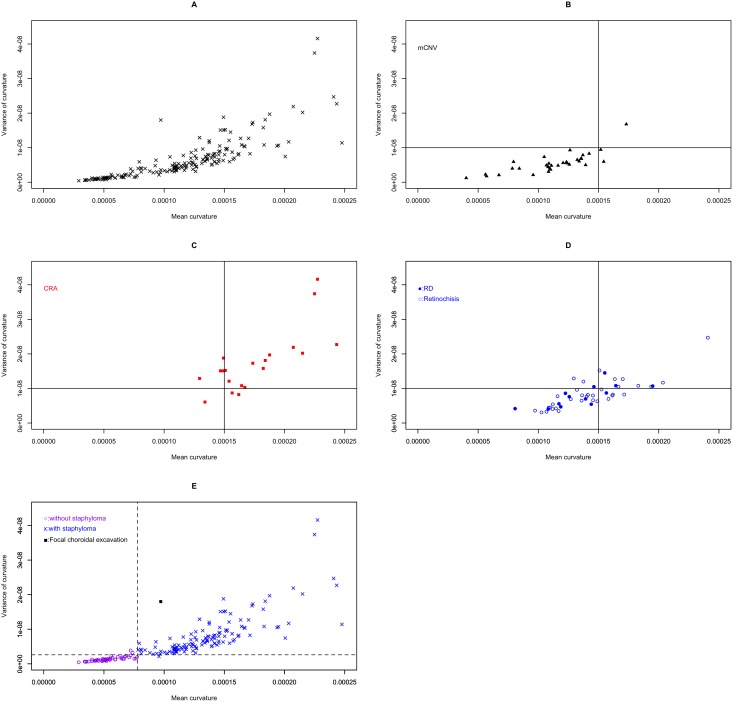
Scatter plot assigning mean curvature to the horizontal axis and variance of curvature to the vertical axis. **A,** All 182 highly myopic eyes are plotted. **B,** 33 eyes with myopic choroidal neovascularization (mCNV) are highlighted. Most values localize to the lower segment of the splitting line at 0.00015 for mean curvature and 1×10^−8^ for variance of curvature. **C,** 17 eyes with severe chorioretinal atrophy (CRA) are highlighted. Most of these values localize to the higher segment of the splitting line. **D,** 33 eyes with retinoschisis (open circles) and 11 eyes with foveal retinal detachments (filled circles) are plotted. **E,** Eyes with and without staphylomas. The groups are easily separated into 2 groups by broken lines that indicate 0.000078 in mean curvature and 0.26×10^−8^ in curvature variance. One eye without a staphyloma had focal choroidal excavation (square).

When we evaluated the color maps for 182 eyes, we found roughly 3 patterns of curvature maps: a yellow dominant curvature map (Figure 3C), a map with an orange-red color at the center (Figure 3A and 3B), and one with a mosaic pattern of red and green (Figure 3D). The comparison between eyes with yellow-dominant curvature maps and eyes that yielded maps with an orange-red color at the center suggested that the degree of staphyloma could be represented by the mean curvature value. In addition, the mosaic patterns of red and green suggested that the smoothness of the staphyloma surface could be represented by the variance in curvature. Hence, we calculated the mean absolute curvature and variance of curvature for each eye as an index for fundus shape. This approach allowed for a quantitative evaluation of the characteristics of fundus shape. With mean curvature values assigned to the horizontal axis and the variance values assigned to the vertical axis, a scatter plot was used to illustrate the consecutive distribution of all eyes evaluated (Figure 4A).

### Characteristics of fundus curvature in eyes with myopic complications

Scatter plots showed that each myopic complication evaluated had a specific profile, as represented by mean curvature and variance. Eyes with mCNV exhibited low-to-moderate levels of curvature and minimal variance (Figure 4B). In contrast, eyes with severe CRA exhibited higher levels of both curvature and variance (Figure 4C). When we drew a splitting line at 1.5×10^−4^ for mean curvature and 1×10^−8^ for the variance of curvature, most eyes with mCNV were distributed throughout the left-lower area, while most eyes with CRA were distributed throughout the right-upper area. Eyes with RD and retinoschisis were similar in exhibiting moderate levels of both curvature and variance (Figure 4D).

The quantitative evaluation of mean curvature and variance of curvature revealed additional differences in fundus shape that distinguished each of the complications studied from the others. Mean curvature was significantly larger in eyes with complications than in eyes without any of the complications studied (Table 2). When the values of eyes with RD and eyes with retinoschisis were combined for further analysis, mean curvature values were significantly smaller in eyes with mCNV as compared to eyes with RD or retinoschisis, and significantly smaller in eyes with RD or retinoschisis as compared to eyes with CRA. Similar to the mean curvature results, variance of curvature was significantly larger in eyes with retinoschisis, RD, and CRA than in eyes without complications (Table 3). However, there was no significant difference in the variance of curvature between eyes with mCNV and eyes without complications. Among the eyes with complications, the variance of curvature also showed significant trends: smaller in eyes with mCNV, intermediate in eyes with RD or retinoschisis, and larger in eyes with CRA.

**Table 2:** Mean absolute curvature of eyes with each complication.

Complication	No complications	mCNV	Retinoschisis	RD	CRA
value (x10^5^: mean ± SD)	8.61±4.27	11.27±3.01	14.39±3.17	13.64±2.89	17.54±3.28
No complications	-	0.005	<0.001	<0.001	<0.001
mCNV	-	-	0.006	0.29	<0.001
Retinoschisis	-	-	-	0.97	0.02
RD	-	-	-	-	0.03

P-values* are shown.

*Tukey-Kramer’s multiple comparison.

CNV: choroidal neovascularization, RD: retinal detachment, CRA: chorioretinal atrophy.

**Table 3:** Mean variance of curvature for eyes with each complication.

Complication	No complications	mCNV	Retinoschisis	RD	CRA
value (x10^9^: mean ± SD)	3.37±3.08	5.53±2.87	8.42±4.29	7.87±3.15	17.40±8.88
No complications	-	0.10	<0.001	0.004	<0.001
mCNV	-	-	0.047	0.45	<0.001
Retinoschisis	-	-	-	0.99	<0.001
RD	-	-	-	-	<0.001

P-values* are shown.

*Tukey-Kramer’s multiple comparison.

CNV: choroidal neovascularization, RD: retinal detachment, CRA: chorioretinal atrophy.

### Characteristics of fundus curvature in eyes with staphylomas

Of the 182 eyes evaluated, 129 eyes were judged to have staphylomas, while 52 eyes were not. One eye without staphyloma had focal choroidal excavation, which was visualized clearly as a red spot on the color map. When the eye with focal choroidal excavation was excluded from the analysis, the highly myopic patients with staphylomas were found to be older than those without. There were also more women than men with staphylomas (Table 4). Furthermore, eyes with staphylomas had significantly greater axial length, mean curvature, and variance of curvature. Figure 4E shows the distribution of eyes with staphylomas vs. those without in the scatter plot of mean curvature and variance of curvature. When we drew a watershed line at 7.8×10^−5^ for mean curvature and 0.26×10^−8^ for variance of curvature, 98.4% of eyes with staphyloma were distributed in areas of the plot corresponding to greater curvature and greater variance, while 96.2% of eyes without staphylomas aggregated to the zones of low curvature and low variance.

**Table 4:** Characteristics of the included eyes according to the presence of staphylomas.

Variable	without staphyloma	with staphyloma	P-value*
n	52	129	
Age (years ± SD)	49.4±12.5	65.7±12.1	<0.01
Sex (male:female)	29∶23	33∶96	<0.01†
Axial length (mm ± SD)	27.34±1.08	29.24±1.66	<0.01
Mean absolute curvature (x10^5^: mean ± SD)	5.36±1.24	13.71±3.50	<0.01
Variance of curvature (x10^9^: mean ± SD)	1.35±0.65	8.26±6.03	<0.01

*unpaired *t*-test.

† Fisher’s exact test.

## Discussion

In the current study, we successfully visualized the shape of the posterior pole by using a novel method to analyze the curvature of 12 lines of 9-mm radial OCT scans. The color map of curvature effectively localized the staphyloma itself as well as the surrounding border. Furthermore, mean curvature and variance of curvature calculations were used to quantitatively evaluate fundus shape, which revealed associations between myopic complications and fundus shape. Our findings suggest that mean curvature and variance of curvature values could be used to define staphylomas and quantitatively evaluate staphylomas.

At present, the process of mCNV development has not been fully elucidated. Lacquer cracks and CRA often lead to mCNV in highly myopic eyes. [23] Curtin and Karlin showed that axial length was associated with the development of lacquer cracks and CRA in high myopia. [24] They also showed that the development of mCNV was not dependent on increases in axial length. Our study also showed that axial length did not differ significantly between highly myopic eyes with mCNV and highly myopic eyes without myopic complications.

The analysis of posterior fundus shape could provide valuable insights for mCNV development. Our findings suggest that mCNV develops in eyes with moderate curvature without undulation. Considering that mCNV was observed in eyes with staphyloma as well as in eyes without staphyloma, the presence of a staphyloma itself was not able to predict the development of mCNV in this study. Previous reports on the relationship between staphyloma and the development of mCNV were inconsistent. Although 2 reports graded staphyloma using B-scan ultrasonographic images, one study reported that the prevalence of mCNV was not associated with staphyloma grade, [13] while another reported that mCNV development was inversely correlated with staphyloma severity. [25] In contrast to these reports using B-scan ultrasonographic images, a study using OCT images reported a direct correlation between mCNV development and staphyloma severity. [18] Quantitative evaluations of the entire posterior pole should elucidate the relationship between eye shape and mCNV development.

In contrast to eyes with mCNV, eyes with severe CRA tended to have severe staphylomas with undulated surfaces. Considering that patchy and diffuse CRA are reported to predispose the patient to the development of mCNV [23], [26] while CRA also develops in the area proximal to regressed mCNV, [27], [28] it might be expected that eyes with mCNV and eyes with CRA would exhibit similar shape. The observed differences in mean curvature and variance of curvature could be explained by the fact that severe CRA can develop after the inactivation of mCNV, causing concave deformation in the posterior pole [29]. The imaging strategy outlined in this study could be used to capture such temporal changes in fundus shape. The evaluation of time-course changes in fundus shape and the association with mCNV and CRA development might elucidate a causal relationship between mCNV and CRA. Such analysis might also identify factors that can be used to predict progression from mCNV to CRA, or vice versa.

The present study could not detect significant differences in fundus shape between eyes with retinoschisis and eyes with RD. The number of eyes with retinoschisis or RD may not have been sufficient for statistical evaluation. Since RD in highly myopic eyes are reported to originate from retinoschisis [30] while retinoschisis does not always lead to RD, the ability to predict RD development from retinoschisis could prevent severe visual loss in highly myopic eyes. Currently, factors such as age, refractive error, axial length, staphyloma, CRA, and vitreoretinal interface quality are hypothesized to play a role in progression from retinoschisis to RD, [31], [32] but the mechanisms involved have not been thoroughly elucidated. Further quantitative study of fundus shape might elucidate mechanisms of the progression form retinoschisis to RD.

Our methodology also allows for the quantification of staphyloma severity. Using the technology described here, staphylomas could be graded objectively on the basis of mean curvature and variance of curvature. Furthermore, our method might be able to distinguish eyes with staphyloma and eyes without staphyloma by the numerical values of mean curvature and variance of curvature. We may be able to define staphyloma by cut-off value of such parameters as well as high myopia by spherical equivalents of ≤−5D, ≤−6D, or ≤−8D, or axial length of ≥26.0 mm, ≥26.5 mm, or ≥28.0 mm. Similarly, mean curvature and variance of curvature could objectively define staphyloma, and further study should verify the value of mean curvature as >7.8×10^−5^ and the mean variance of curvature as >0.26×10^−8^ used in this study. Currently, staphylomas are usually graded in a subjective manner based on slit-lamp biomicroscopy. [12] Our method with wider OCT could be used to elaborate a standardized system for objective staphyloma classification.

This study has certain limitations. One is a segmentation problem. The auto-segmentation used for this research is accurate when studying the normal eye but not as well when studying highly myopic eyes, because the OCT images obtained in the latter case are often of poor quality. Manual segmentation support was therefore used in such cases presented here. The second issue involves scan width. The 9-mm scan width employed for this study is actually wider than that used in previous studies. However, it was not sufficiently wide to include the entire staphyloma border in all cases. We could detect the entire staphyloma edge only in 4 eyes out of 129 eyes with staphyloma. Third, this study utilized a cross-sectional study design using case series, which precluded causational analysis. Future studies should measure changes in curvature and variance over time. Associations between myopic complications and posterior fundus curvature should be further examined in high myopic patients without subjective complaint or myopic but not highly myopic eyes. Fourth, in a strict sense, the lines we plotted may not simply be attributed to Bruch’s membrane line. Although fourth hyperreflective line in OCT is generally recognized as retinal pigment epithelium and the line we plotted as Bruch’s membrane, [33], [34] Spaide and Curcio discussed that the origin of the fourth hyperreflective line in OCT remained to be determined. [35] Lastly, the current study is evaluating the curvature of OCT images, not the true curvature of the eyeball. As being reported by Kuo et al., [36] the OCT images were more flattened than MRI images. Indeed, as shown in the Supplemental Note, Bruch’s membrane line in OCT becomes considerably steeper after correction. However, since we evaluated the local curvature by every 1,000-µm distance rather than by eye shape as a whole, the curvature map did not notably change after correction (Details are shown in Supplemental Note). For the clinical setting, evaluating OCT images is more practical than evaluating true eye shape, from the viewpoint of accessibility. Considering the minor effect of correction to the local curvature evaluation, it would be more helpful for ophthalmologist to use the OCT image without correction.

Despite these limitations, this novel quantitative approach allowed us to correlate fundus shape and myopic complications. Furthermore, we demonstrated how these maps could be used to evaluate fundus shape. The ability to evaluate staphylomas quantitatively will facilitate investigations into the associations between myopic complications and staphylomas.
